# Chromosome Synapsis and Recombination in Male-Sterile and Female-Fertile Interspecies Hybrids of the Dwarf Hamsters (*Phodopus*, Cricetidae)

**DOI:** 10.3390/genes9050227

**Published:** 2018-04-25

**Authors:** Tatiana I. Bikchurina, Katerina V. Tishakova, Elena A. Kizilova, Svetlana A. Romanenko, Natalya A. Serdyukova, Anna A. Torgasheva, Pavel M. Borodin

**Affiliations:** 1 Institute of Cytology and Genetics, Russian Academy of Sciences, Siberian Department, Novosibirsk 630090, Russia; Bikchurina@bionet.nsc.ru (T.I.B.); 95katerina95@mail.ru (K.V.T.); pinus@bionet.nsc.ru (E.A.K.); torgasheva@bionet.nsc.ru (A.A.T.); 2 Novosibirsk State University, Novosibirsk 630090, Russia; rosa@mcb.nsc.ru; 3 Institute of Molecular and Cellular Biology, Russian Academy of Sciences, Siberian Department, Novosibirsk 630090, Russia; serd@mcb.nsc.ru

**Keywords:** hybrid sterility, sex chromosomes, pseudoautosomal region, synaptonemal complex, MLH1, SYCP3, γH2A.X

## Abstract

Hybrid sterility is an important step in the speciation process. Hybrids between dwarf hamsters *Phodopus sungorus* and *P.* *campbelli* provide a good model for studies in cytological and genetic mechanisms of hybrid sterility. Previous studies in hybrids detected multiple abnormalities of spermatogenesis and a high frequency of dissociation between the X and Y chromosomes at the meiotic prophase. In this study, we found that the autosomes of the hybrid males and females underwent paring and recombination as normally as their parental forms did. The male hybrids showed a significantly higher frequency of asynapsis and recombination failure between the heterochromatic arms of the X and Y chromosomes than the males of the parental species. Female hybrids as well as the females of the parental species demonstrated a high incidence of centromere misalignment at the XX bivalent and partial asynapsis of the ends of its heterochromatic arms. In all three karyotypes, recombination was completely suppressed in the heterochromatic arm of the X chromosome, where the pseudoautosomal region is located. We propose that this recombination pattern speeds up divergence of the X- and Y-linked pseudoautosomal regions between the parental species and results in their incompatibility in the male hybrids.

## 1. Introduction

Accumulation of genetic differences during an independent evolution of geographically isolated populations leads to incompatibility between parental genomes. Hybrid sterility is the most common symptom of such incompatibility. Analysis of the initial steps of hybrid sterility is important for understanding the genetic and cytological mechanisms of speciation [1,2]. Studies on mammalian hybrids indicated abnormalities in meiotic chromosome pairing as a common cause of hybrid sterility [3,4,5,6]. They also confirmed the validity of Haldane’s rule stating that signs of sterility occur earlier and are stronger in hybrids of the heterogametic sex than in the homogametic sex [7]. Male hybrid sterility is often accompanied by pairing and recombination failure in the pseudoautosomal region (PAR), a short genome region retaining homology between the X and Y chromosomes [8,9,10,11,12].

Hybrids of the dwarf hamsters *Phodopus sungorus* and *P. campbelli* (Cricetidae, Rodentia) provide an interesting model for studying the genetic and cytological mechanisms of hybrid sterility. The parental species diverged about one million years ago [13] and retained almost identical karyotypes: the same diploid chromosome number (28) and number of autosome arms (51). They show minor differences in size and location of C-heterochromatin blocks at some chromosomes, but both species have large heterochromatic arms at X and Y chromosomes [14,15,16]. Yet, the interspecies hybrids show reduced fitness, although the ways of the fitness reduction differ between and within the reciprocal crosses. The hybrids produced in the crosses of *P. sungorus* dams to *P. campbelli* sires show extensive placental and embryonic growth, which led to high late-term embryonic and maternal mortality rates [17]. The crosses of *P. campbelli* dams to *P. sungorus* sires produce offspring of normal body size. Breeding tests show the normal fertility of the hybrid females, while the hybrid males are completely sterile. They have small testes and show various abnormalities of spermatogenesis. In some of them, meiosis does not start at all. In some, spermatogenesis is arrested at postmeiotic stage. In some, it proceeds to term, but is accompanied by massive apoptosis of primary spermatocytes and results in abnormal sperm severely reduced in count and motility [11,18]. Several studies of the cytological basis of sterility detected a high frequency of asynapsis between the X and Y chromosomes in hybrid males and suggested this particular abnormality as the main cause of sterility [11,19,20,21]. However, some important aspects of meiotic chromosome behavior in dwarf hamster hybrids remained unknown. Chromosome synapsis in female hybrids have not been studied yet. Nothing is known about recombination frequency and distribution at the sex chromosomes and autosomes in male and female hybrids.

In this study, we carried out a histological analysis of the dynamics of spermatogenesis in the hybrids between *P. campbelli* (dams) and *P. sungorus* (sires) and in the parental species. In the male hybrids that showed meiotic progression we examined synapsis and recombination of autosomes and sex chromosomes and compared their meiotic characteristics with those of female hybrids on the one hand and males and females of the parental species on the other hand. We visualised chromosome synapsis using immunolocalisation of SYCP3, the protein of the lateral elements of the synaptonemal complex (SC). Immunolocalisation of MLH1, a mismatch repair protein marking mature recombination nodules [22], allowed us to assess the overall recombination rate in the hybrids and parental species and to generate karyotype-specific recombination maps for the sex chromosomes and several autosomes. Antibodies to γH2A.X—the phosphorylated form of histone H2A.X—were used as markers of unrepaired DNA double-strand breaks (DSBs) and transcriptionally silenced chromatin [23].

## 2. Materials and Methods

### 2.1. Animals

Outbred laboratory colonies of the parental species were maintained via outcrossing between families in the animal house of the Institute of Cytology and Genetics. The hybrids were the progeny of crosses between *P. campbelli* (dams) and *P. sungorus* (sires). Maintenance, handling and euthanasia of animals were carried out in accordance with the approved national guidelines for the care and use of laboratory animals. All experiments were approved by the Ethics Committee on Animal Care and Use of the Institute of Cytology and Genetics of the Siberian Department of the Russian Academy of Sciences (approval No. 35 of 26 October 2016).

### 2.2. Histological Analysis

The testes of adult males were removed, separated from *tunica albuginea* and fixed in Bouin’s solution for 48 h, dehydrated in a graded ethanol series, immersed in xylene and finally embedded in paraffin. The testes were sectioned at a thickness of 10 μm and mounted on slides. The sections were deparaffinised, stained with hematoxylin and eosin and examined at low and high magnifications under an Axioscop 2 plus microscope (Carl Zeiss, Jena, Germany) equipped with a CCD camera AxioCam HRc (Carl Zeiss) and AxioVision image-processing package (Carl Zeiss). Only tubules of strictly frontal section were taken into the analysis. The stages of the seminiferous epithelium cycle were determined at the cross-sections of the testis according to Leblond and Clermont [24] and Wing and Christensen [25]. The ratios of various cell types of spermatogenic epithelium were estimated in the tubules at stages VI–VIII. Caudal epididymitis was minced in the phosphate buffered saline (PBS) at 37 °C. The sperm morphology was examined at the smear prepared on glass slides. Sperm abnormalities were classified according to Wyrobek and Bruce [26].

### 2.3. Synaptonemal Complex Spreading and Immunostaining

Chromosome spreads were prepared from the testes of adult males and the ovaries of newborn females by air-dried method [27]. For electron microscopic examination, the spreads were stained with silver nitrate [26] and covered with a plastic film. After light microscopic examination, the spreads were transferred to specimen grids and examined under an electron microscope JEM-1400 (JEOL, Tokyo, Japan) at 80 kV. Immunostaining was performed according to the protocol described by Anderson et al. [22] using rabbit polyclonal anti-SYCP3 (1:500; Abcam, Cambridge, UK), mouse monoclonal anti-SYCP3 (1:100; Abcam), mouse monoclonal anti-MLH1 (1:30; Abcam), rabbit polyclonal anti-γH2A.X (1:330; Abcam) and human anticentromere (ACA) (1:70; Antibodies Inc., Davis, CA, USA) primary antibodies. The secondary antibodies used were Cy3-conjugated goat anti-rabbit (1:500; Jackson ImmunoResearch, West Grove, PA, USA), fluorescein isothiocyanate (FITC)-conjugated goat anti-mouse (1:30; Jackson ImmunoResearch) and aminomethylcoumarin (AMCA)-conjugated donkey anti-human (1:40; Jackson ImmunoResearch) antibodies. Antibodies were diluted in PBT (3% bovine serum albumin and 0.05% Tween 20 in PBS). A solution of 10% PBT was used for blocking. Primary antibody incubations were performed overnight in a humid chamber at 37 °C; and secondary antibody incubations, for 1 h at 37 °C. Slides were mounted in Vectashield antifade mounting medium (Vector Laboratories, Burlingame, CA, USA) to reduce fluorescence fading. The preparations were visualized with an Axioplan 2 microscope (Carl Zeiss) equipped with a CCD camera (CV M300, JAI Corporation, Yokohama, Japan), CHROMA filter sets and ISIS4 image-processing package (MetaSystems GmbH, Altlußheim, Germany). The location of each imaged immunolabelled oocyte spread was recorded so that it could be relocated on the slide after fluorescence in situ hybridization (FISH).

### 2.4. Fluorescence In Situ Hybridization

After acquisition of the immunofluorescence signals, the oocyte preparations were subjected to FISH with a golden hamster X chromosome paint probe generated in the Key Laboratory of Cellular and Molecular Evolution, Chinese Academy of Sciences, by degenerate oligonucleotide priming (DOP)-PCR amplification of flow-sorted chromosomes. FISH was performed using a standard protocol [28]. Briefly, 15 μL of hybridization buffer contained 0.2 μg of the biotinylated DOP-PCR product, 2 μg of Cot-10 DNA of *P. campbelli* [29], 10% dextran sulfate, 40% formamide in 2× SSC (saline-sodium citrate buffer). Paints were denatured in 70% formamide in 2× SSC for 1 min at 60 °C and reannealed for 1 h at 42 °C. Probes were hybridized overnight at 42 °C. Posthybridization washes included 2× SSC, 0.4× SSC, 0.2× SSC (5 min each, 45 °C) followed by 20-min incubation in 3% dry milk in 4× SSC/0.5% Triton X-100. All washes were performed at 45 °C in 4× SSC/0.5% Triton X-100 3 times (5 min each). Hybridization signals were detected with avidin-FITC without further amplification.

### 2.5. Image Analysis

MLH1 signals were scored only if they were localised on the SC. The length of the SC of all bivalents was measured using MicroMeasure 3.3. [30]. Bivalents 1, 4, 5 and 7 were identified by their size and centromere index (CI). The XY bivalent was recognised by the presence of the unpaired differentiated part of the X chromosome. The XX bivalent was identified by FISH with the golden hamster X chromosome. To generate recombination maps of the identified chromosomes, we calculated the absolute position of each MLH1 focus multiplying the relative position of each focus by the absolute length for the appropriate chromosome arm averaged for sex and group (the parental species and the F1 hybrids are called groups throughout). These data were pooled for each arm and graphed to represent a recombination map.

Statistica 6.0 software package (StatSoft, Tulsa, OK, USA) was used for descriptive statistics. To compare SC length and MLH1 focus number between parental species and their F1 hybrids, Student’s *t* test (two-sided) and Mann–Whitney *U* test (two-sided) were performed. Homogeneity of the samples was tested by Kruskal–Wallis *H* test. MLH1 focus distributions along the same bivalents were compared using Kolmogorov–Smirnov test. χ^2^ test was used to test the difference in the frequency of various cell fractions.

## 3. Results

### 3.1. Spermatogenesis

The breeding test indicated complete fertility of all examined males of the parental species. Each of them produced at least three litters in between-family crosses. None of the hybrid males produced a progeny in at least three matings with the fertile females of the parental species. The average testis mass was significantly lower in the hybrids (0.255 ± 0.182 g) compared to *P. sungorus* (0.727 ± 0.024 g) and *P. campbelli* (1.158 ± 0.021 g) (Mann–Whitney *U* test, *p* < 0.01). However, there was a polymorphism in the testis mass among the hybrids. Three males of one family showed a significantly lower mass (0.035 ± 0.002 g) than the other hybrids (0.387 ± 0.082 g) (*p* < 0.05). Hereafter, we call them type A and type B hybrids, correspondingly.

**Figure 1:**
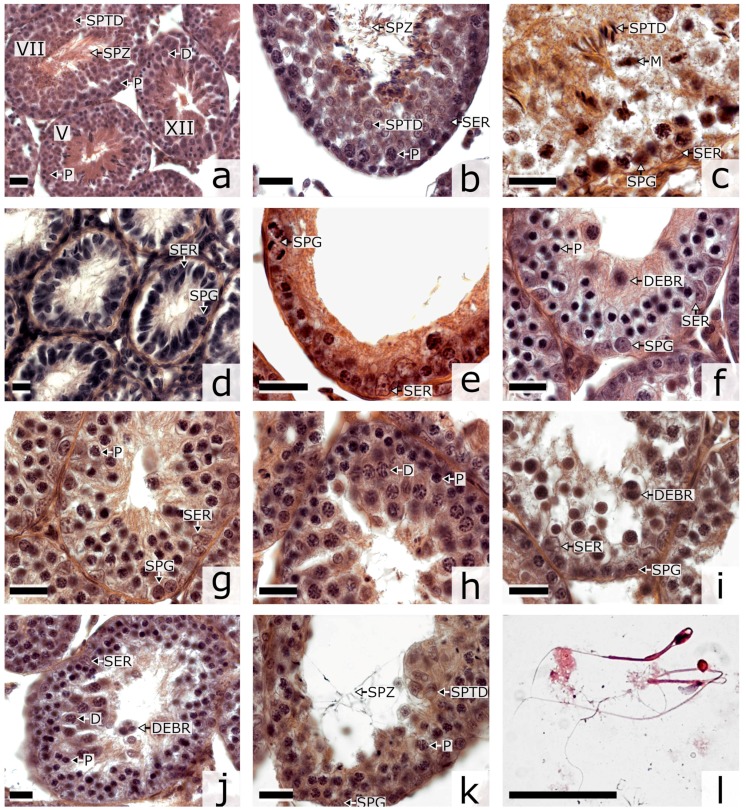
Histological sections of testes of *Phodopus campbelli* (**a**–**c**) and F1 type A (**d,e**) and type B (**f**–**l**) hybrids stained by hematoxylin-eosin. D, diplotene-diakinesis; DEBR, cellular debris; M, metaphase I and II; P, pachytene; SER, Sertoli cell; SPG, spermatogonium; SPTD, spermatid; SPZ, spermatozoid; E, early spermatocyte I at leptotene-zygotene. (**a**) tubules at different stages of the seminiferous epithelium cycle (V, VII and XII) showing an undisturbed progression of the spermatogenic wave; (**b**) stage VIII tubule containing Sertoli cells, spermatocytes I at pachytene and spermatids, its lumen containing mature spermatozoa; (**c**) stage XIII-XIV tubule containing spermatocytes at metaphase I and II and spermatids at the upper layer and Sertoli cells and spermatogonia at the lower layer; (**d**) aberrant seminiferous tubules without spermatogenic epithelium, (**e**) wall of an empty tubule with dividing spermatogonia and Sertoli cells only; (**f**) tubule with an excess of early spermatocytes I at leptotene and zygotene at the lower layer, cellular debris at the upper layer; (**g**) tubule with an excess of spermatocytes I at pachytene; (**h**) tubule wall with an excess of spermatocytes I at diplotene-diakinesis; (**i**) tubule containing degenerating pachytene spermatocytes and cellular debris; (**j**) tubule containing degenerating diplotene-diakinesis spermatocytes and cellular debris; (**k**) tubule with abnormal spermatids in the wall and abnormal spermatozoa in the lumen; (**l**) abnormal spermatozoa in the epididymal smear. Bar: 20 µm.

To gain insight into the causes of male hybrid sterility, we carried out histological analysis of the dynamics of the seminiferous epithelium in the parental species (Figure 1a–c) and the hybrids (Figure 1d–k). The hybrids showed multiple aberrations in the morphology of the seminiferous tubules and their contents. Many of the tubules had a dilated lumen and a disorganized seminiferous epithelium cycle (Figure 1d–j). The layered structure of spermatogenic epithelium was severely disrupted (Figure 1e–j). The lumen of the convoluted tubules was usually either dilated (Figure 1g–j) or empty (Figure 1e,f,k). It contained no normal maturing or mature spermatozoa. Rare spermatids and single spermatozoa of abnormal morphology were observed in type B hybrids (Figure 1j,k). Especially severe defects were observed in type A hybrids. Their tubules did not contain spermatogenic epithelium. Histological examination detected very rare spermatogonia and numerous Sertoli cells (Figure 1d). These animals were not suitable for further cytological analysis.

Compared to the parental species, the type B hybrids showed many more tubules with depleted spermatogenic epithelium (Figure 1d,e) and tubules at stages XII–XIV containing intact and degenerating pachytene—diplotene spermatocytes (Figure 1f–h) (Student’s *t* test, *p* < 0.001). Also, these hybrids had many fewer tubules at stages VI–XI containing maturing and mature spermatozoa (*p* < 0.001) (Table 1). The ratio of pachytene spermatocytes (P) to spermatogonia (SPG) in the hybrids was two times higher than that in the males of the parental species (Student’s *t*-test, *p* < 0.001). At the same time, we observed a severe shortage of postmeiotic cells: early and middle spermatids (SPTD) (*p* < 0.001) (Table 1).

Caudal parts of epididymes in the hybrids were underdeveloped, translucent and contained very little fluid, whereas in the parental specimens they were opalescent and filled with seminal fluid. Spermatozoa were very rare at the epididymal smears of the F1 hybrids, and abundant at the smears of the parental species (Figure S1). Almost all spermatozoa of the hybrids showed multiple abnormalities (Figure 1l, Figure S1c–e). Spermatozoa of the parental species showed isolated rather than multiple abnormalities and in much lower frequency (Figure S1a,b, Table S1).

### 3.2. Autosome Pairing and Recombination

We identified meiotic prophase substages by immunolocalization of SYCP3, γH2A.X and MLH1 (Figure S2). Leptotene cells contained short patches of SC surrounded by disperse clouds of γH2A.X-labelled chromatin. At zygotene, we observed partial or complete asynapsis, heterosynapsis and interlocking of autosomes and sex chromosomes. Unpaired chromosome regions were densely labelled with γH2A.X. The onset of pachytene was marked by complete synapsis of the autosomal SCs, predominant localisation of γH2A.X at the sex bivalent and its focal occurrence at autosomes. At mid and late pachytene cells, we observed MLH1 foci at most autosomes, while γH2A.X was present at the sex body only. Non-homologous associations and interlockings were extremely rare at this substage (Figures S2 and S3, Table S2). Diplotene was characterized by the disappearance of MLH1 foci and gradual desynapsis of the autosomes.

**Figure 2:**
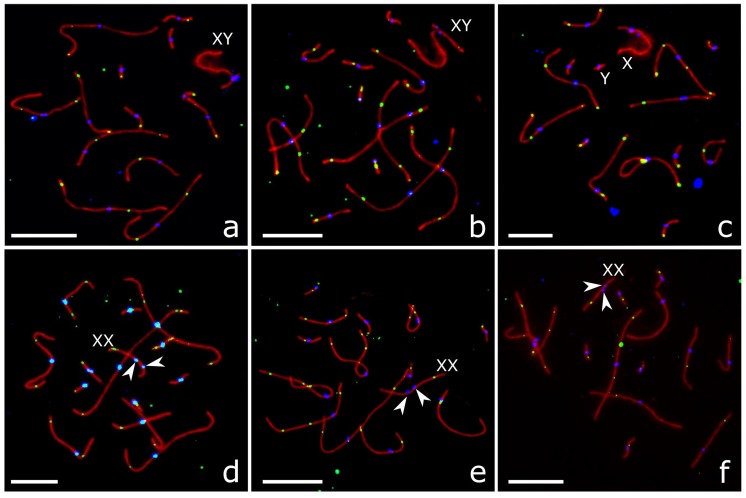
Pachytene spermatocytes (**a**–**c**) and oocytes (**d**–**f**) of *P. sungorus* (**a**,**d**), *P. campbelli* (**b**,**e**) and F1 hybrids (**c**,**f**) after immunolocalisation of SYCP3 (red), MLH1 (green) and centromeric proteins (blue). Arrowheads show misaligned centromeres at XX bivalents. Bar: 10 µm.

For our analysis, we selected cells at the mid-late pachytene marked by the occurrence of MLH1 foci at most autosomes (Figure 2).

Table 2 shows prominent sex differences in the total length of the autosomal SC. Like in any other mammalian species, females of the dwarf hamsters have significantly longer SCs than males (Student’s *t* test, *p* < 0.01 for each group). Within each sex, the differences between the hybrids and parental species were not significant (*p* > 0.05).

There was a significant individual variation in MLH1 focus number within the parental species both in males and females (Kruskal–Wallis *H* test *p* > 0.01). The hybrid males and females were rather homogeneous for this trait (*p* > 0.05). We did not detect significant differences in MLH1 focus number between hybrids and parental species (Table 2; Mann–Whitney *U* test, *p* > 0.05). Thus, the hybrids do not show a reduction in overall recombination rate.

We estimated recombination characteristics in four individual autosomal bivalents that were unequivocally identifiable by their length and centromeric index (Table S3). A comparison of the bivalents revealed a significant sex difference in their length (Student’s *t* test after Bonferroni correction for multiple comparisons, *p* < 0.00001) and no sex difference in MLH1 focus number within each group (*p* > 0.004). The hybrids of both sexes did not show any significant difference from the parental species in the MLH1 focus number at each bivalent examined (*p* > 0.003) (Table 2).

**Figure 3:**
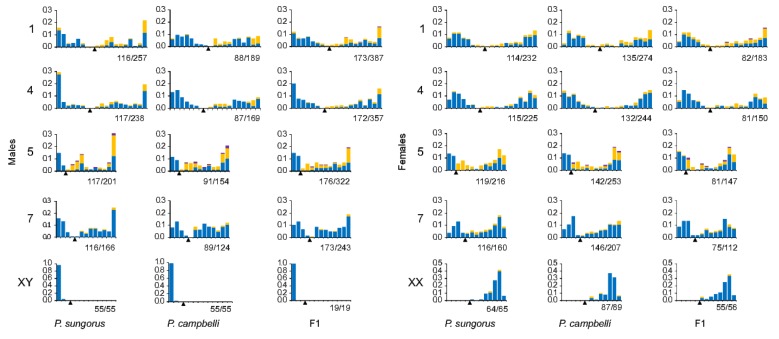
Distribution of MLH1 foci along identifiable bivalents from pachytene spermatocytes and oocytes of *P. sungorus*, *P. campbelli* and F1 hybrids. The bivalents are indicated on the left-hand side of the graphs. Centromere positions are shown by arrowheads. The X axis shows the position of MLH1 foci at the bivalent. The marks on the X axis are separated by approximately 1 µm of the SC length. Stacked columns show the frequency of the bivalents containing one (blue), two (yellow) and three (violet) MLH1 foci within each interval. The figures in the legends below each plot show the number of chromosomes/MLH1 foci plotted.

The patterns of MLH1 distribution in the hybrids and parental species were similar to those described for other mammals [31,32]. The frequency of MLH1 foci was high near the distal chromosome ends and low around the centromeres. Bivalents showed a more even distribution in oocytes than in spermatocytes (Figure 3). We did not detect significant differences between the parental species and F1 hybrids of the same sex in MLH1 focus distribution along the same bivalents (Kolmogorov–Smirnov test, *p* > 0.003).

### 3.3. XY Chromosome Pairing and Recombination

**Figure 4:**
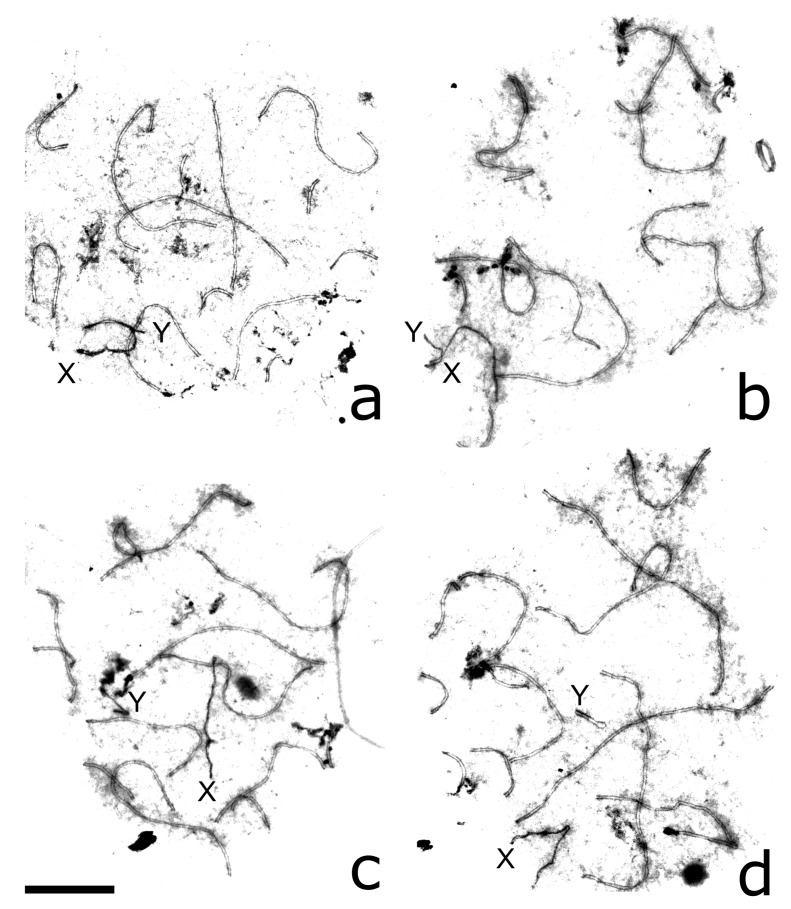
Electron microphotographs of pachytene spermatocytes of *P. sungorus* (**a**) and F1 hybrids (**b**–**d**) after AgNOR staining. Letters indicate the distal ends of the sex chromosome axes. (**a**) complete synapsis between Xp and Yq; (**b**) association between misaligned ends of Xp and Yq; (**c**) asynapsis of X and Y; (**d**) asynapsis of *X* and *Y* with the Y axis forming self-synapsis fold-back. Bar: 10 µm.

**Figure 5:**
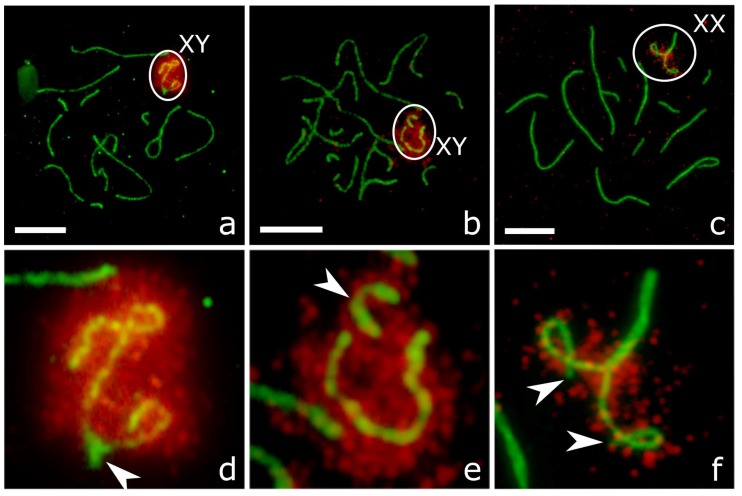
Pachytene spermatocytes (**a**,**b**) and oocyte (**c**) of *P. sungorus* (**a**) and F1 hybrids (**b**,**c**) after immunolocalisation of SYCP3 (green), and γH2A.X (red). Bottom panel (**d**–**f**) shows zoom of the upper white ellipses. (**a**,**d**) completely synapsed XY bivalent. Asynapsed parts of Xq and Yp are labelled with γH2A.X. Arrowhead indicates the unlabelled pairing region; (**b**,**e**) co-localised asynapsed X and Y axes in a shared cloud of γH2A.X-positive chromatin. Arrowhead indicates Y axis; (**c**,**f**) XX bivalent with asynapsed Xp. Arrowheads indicate the ends of asynapsed axes. Bar: 10 µm.

Most pachytene cells of *P. sungorus* and *P. campbelli* contained partially or completely synapsed XY bivalents (Figure 2a,b, Figure 4a and Figure 5a,d; Table 3). Synapsis involved distal parts of the short arm of X (Xp) and long arm of Y (Yq). In some cells, Yp was completely paired with the proximal part of Xq. The distal part of Xq remained unpaired and showed heavy γH2A.X labelling, while the pairing region involving Xp and Yq appeared unlabeled (Figure 5a,d).

In the hybrids, the majority of pachytene spermatocytes contained loosely associated (Figure 4b) or completely asynapsed axes of the X and Y chromosomes (Figure 2c, Figure 4c,d and Figure 5b,e). In some cells, the unpaired Y axis formed fold-backs of self-synapsis (Figure 4d). Asynapsed sex chromosomes were uniformly labelled with γH2A.X along their entire length (Figure 5b,e; Table 3).

The difference between the parental species and hybrids in the frequency of XY asynapsis was significant (χ^2^ test, *p* < 0.0001). The asynapsed X and Y axes were localised in the common nuclear compartment surrounded by a shared cloud of γH2A.X-positive chromatin in about 80% of cells with XY asynapsis (Figure 5b,e). In the remaining cells, we observed the X and Y axes at a distance from each other (Figure 4c,d).

A large fraction of XY bivalents lacked MLH1 foci in all groups (Table 3). The differences in the frequency of such bivalents between parental species on the one hand and between the hybrids and *P. campbelli* on the other hand were significant (χ^2^ test, *p* = 0.0013 and 0.0008, correspondingly), while the difference between the hybrids and *P. sungorus* was not significant (*p* = 0.106) (Table 3). A relatively high frequency of XY bivalents lacking MLH1 foci in all groups might be due to a transitory nature of the foci. In mammals, they usually appear and disappear at the XY earlier than at the autosomal bivalents [22,33,34]. Alternatively, the sex bivalents without MLH1 foci might be achiasmatic. It has been shown that in man the frequency of XY bivalents lacking MLH1 is correlated with the frequency of spermatozoa aneuploid for sex chromosomes [35].

When an MLH1 focus was present at the XY bivalent, it was always located at the pseudoautosomal region (PAR) situated at the ends of Xp and Yq. The size of the PAR was estimated as the relative distance between the Xp (Yq) end and the farthest MLH1 focus and was around 10% of the length of the X chromosome in all three groups: about 1 µm or 10% of Xp (Figure 3).

### 3.4. XX Chromosome Pairing and Recombination

In a substantial proportion of oocytes of all groups we observed one of the middle-sized metacentric bivalents with misaligned centromeres (Figure 2d–f). Such bivalents were absent from the spermatocytes. We hypothesized that it was an XX bivalent. FISH with a whole chromosome painting probe of the X chromosome of the golden hamster *Mesocricetus auratus* [36] confirmed this suggestion. The probe produced a strong specific signal at Xq, while heterochromatic Xp remained unstained (Figure S4). The highest frequency of XX centromere misalignment was detected in the hybrids (Table 3). The difference between the hybrids and both parental species was significant (χ^2^ test, *p* < 0.001).

Some of the XX bivalents with misaligned centromeres demonstrated partial or complete asynapsis of the distal ends of Xp. The unpaired ends were labelled with γH2AX antibodies (Figure 5c,f). This aberration was rather rare in the parental species and affected more than half of pachytene oocytes in the hybrids (Table 3). The difference between the hybrids and the parental species was significant (χ^2^ test, *p* < 0.0001).

We have never observed MLH1 foci at heterochromatic Xp. Single MLH1 foci were detected at Xq only (Figure 2b,d,e and Figure 3). Some XX bivalents lacked MLH1 foci at both arms (Table S3). The pattern of the MLH1 focus distribution along Xq was rather similar to that observed at the arms of the metacentric autosomes of the dwarf hamsters with a peak close to the telomere and a valley near the centromere (Figure 3).

## 4. Discussion

Our histological data confirmed the complete sterility of F1 male hybrids between *P. sungorus* and *P. campbelli* detected in previous studies [11,18,19]. Also, we confirmed a heterogeneity between F1 hybrids of the same crosses in the timing and degree of spermatogenesis arrest described by Ishishita et al. [11]. In type A hybrids, it was arrested before meiotic prophase with a very small number of primary spermatocytes passing this block. The main cause of type B hybrid sterility was meiotic arrest at the end of meiotic prophase followed by massive germ cell death. Admittedly, a comparatively low ratio of spermatogonia to Sertoli cells in the hybrids (Table 1) indicates that premeiotic stages may also be affected in type B hybrids. This variation in the onset of spermatogenetic arrest is probably determined by random and genetic variation within the parental species. Dwarf hamsters are maintained as outbred colonies and therefore are genetically heterogeneous, although the level of their heterogeneity is lower than in most outbred strains of other laboratory rodents [37].

Ishishita et al. [11] proposed that the dissociation between the X and Y chromosomes was the main cause of sterility in the type B hybrids of the dwarf hamsters. We also found a high frequency of dissociation between the X and Y chromosomes (about 80%) in the F1 hybrid, while in the males of the parental species it was eight times lower. These data are in good agreement with the results of previous studies, although we found slightly less contrasting differences between the hybrids and parental species in the frequency of this aberration [11,20]. This minor discrepancy may be determined by the genetic differences between the parental strains used in the hybridization experiments or/and different methods of discrimination between meiotic stages. Another reason of the discrepancy might be the differences in the identification of pachytene cells and their distinction from the cells at earlier stages of the meiotic prophase. Since Moses [38], the synaptic state of the X and Y chromosomes has been used as the main indicator of prophase substage. Obviously, this indicator is not valid for the hybrids due to a high incidence of X and Y dissociation. The occurrence of MLH1 foci at autosomes provides a more reliable marker of mid- and late-pachytene cells [39]. Given that only ~20% of XY bivalents are engaged in synapsis (Table 3), and of these, fewer than half exhibit a MLH1 focus, more than 90% of spermatocytes in F1 hybrids could be achiasmatic.

In contrast to some previous studies [19], we did not detect any serious deviations in autosome synapsis and recombination in male and female F1 hybrids compared to the parental species. For these reasons, disturbances of homologous chromosome recognition due to autosome divergence between the parental species appear to be an unlikely cause of hybrid sterility.

Taking X–Y asynapsis as the main cause of male hybrid sterility, we shall discuss two questions. Why does asynapsis lead to meiotic arrest and what is the cause of a high frequency of asynapsis in the hybrids?

The fact that pairing and recombination in the PAR is required for meiotic progression and proper disjunction of the sex chromosomes has been well known for a long time [40,41,42,43]. One of the causes of meiotic failure in the cells with X–Y asynapsis is their inability to pass the pachytene or synapsis checkpoint. Burgoyne et al. [44] demonstrated that synapsis in the PAR is monitored by the checkpoint.

Another cause of detrimental effects of X–Y asynapsis on spermatogenesis might be its interference with meiotic sex chromosome inactivation (MSCI). This process is part of a more general mechanism of meiotic silencing of unsynapsed chromatin (MSUC). In mammalian male meiosis, differentiated parts of the X and Y chromosomes remain unsynapsed and contained unrepaired DSBs. The persistence of DSBs until pachytene induces phosphorylation of histone H2A.X followed by transcriptional inactivation of the unsynapsed parts of the sex bivalent and formation of the XY body. This inactive state is maintained in post-meiotic haploid spermatids [45,46].

Normally, the PAR is completely synapsed at midpachytene. It does not contain unrepaired DSBs and is not usually labelled with antibodies to γH2A.X (see Figure 5a,d) and probably escapes MSCI. It is well known that the genes located in the PAR escape inactivation in female somatic cells [47]. However, their transcription in male meiotic cells is poorly studied, although the expression of several PAR genes such as *Sts* [48] and *Fxy* [49] has been detected in testes. It seems possible that the spreading of MSCI to the PAR and inactivation of the genes essential for spermatogenesis might be a cause of meiotic arrest of the cells with X-Y dissociation. Thus, there are at least two mechanisms by which X–Y asynapsis might result in male hybrid sterility.

The second question is why sex chromosome synapsis is disrupted in the hamster hybrids. The most direct cause of asynapsis in any chromosomal region in the hybrids is the lack of homology. Studies on mouse hybrids indicate that genetic divergence between parental species in the PAR correlates with X–Y dissociation [8,9]. The PAR shows a much higher evolution rate than autosomal or even X chromosome specific genes [50,51]. An especially high rate of PAR evolution has been detected in rodents. Even closely related mouse species differ in PAR genetic content and in the location of the boundary between the PAR and sex chromosome specific regions [49,52]. Many rodent species have apparently evolved an alternative mechanism of X–Y disjunction in meiosis and lost the PAR altogether [10,53,54].

The main cause of rapid PAR evolution is its exceptionally high recombination rate in male meiosis. In humans, the recombination rate in PAR1 is 12.5 times higher in males than in females and 16 times higher than the male genome-wide recombination rate [55,56]. In male mice, the PAR recombination rate is 7-fold greater than that in females [56]. These remarkable differences in the recombination rate are determined by the difference in PAR chromatin organization in male and female meiosis. Kauppi et al. [57] demonstrated that male mouse PAR DNA forms disproportionally long chromosome axes and shorter and more numerous chromatin loops. The expected density of DSBs in the PAR is 10 times higher than that in other genome regions. Such a high recombination rate inevitably results in the genomic instability of the PAR and in increased individual variability for single nucleotide polymorphisms, segmental duplications and copy number variation [58,59].

Male-derived mutations at the PAR might be repaired or segregated during normal female meiosis and then eliminated by natural selection. However, this way of PAR homogenization is completely blocked in the dwarf hamsters. Their Xp arm containing PAR is heterochromatic [14,15,16] and, as we demonstrated in this study, is often asynapsed (Figure 5c,f) or non-homologously synapsed (Figure S4; Table 3) and completely locked for recombination in female meiosis (Figure 3). Suppressive effects of heterochromatin on recombination are well-known [60,61,62]. In the case considered here, recombination suppression is complete. Neither in the hybrid females nor in the females of the parental species had we detected a single XX bivalent with a MLH1 focus at Xp (Figure 3). Recombination block along the entire Xp, including the PAR, should have led to its mutational meltdown and degradation.

Thus, instead of repairing mutations that occurred in the PAR in male meiosis, the XX bivalents apparently accumulate and distribute within the species their own load of PAR mutations. The combination of these two factors—an exceptionally high recombination rate in the XY PAR and no recombination in the XX PAR—accelerates its evolution. Rapid evolution running independently in each species speeds up divergence and loss of homology between their PARs and results in a high incidence of asynapsis in interspecies hybrids, which, in turn, is one of the causes of male hybrid sterility in the dwarf hamsters. Other incompatibilities, such as the nonviability of the reciprocal hybrids and premeiotic sterility of type A hybrids, certainly occurred independently and their mechanisms remain to be examined.

## Figures and Tables

**Table 1:** Relative abundance (±standard deviation (SD)) of seminiferous tubules at different stages of the seminiferous epithelium cycle and the ratios of different cell types in the testes of *Phodopus sungorus*, *P. campbelli* and their F1 hybrids.

Genotype (*n* of Animals)	Stage of Seminiferous Epithelium Cycle			Empty Tubules	*n* of Tubules	Ratio of Cell Types			*n* of Tubules/Cells
	I–V	VI–XI	XII–XIV			SPG/SER ^1^	P/SPG	SPTD/P	
*P. sungorus* (*n* = 4)	0.18 ± 0.01	0.74 ± 0.01	0.07 ± 0.01	0.01 ± 0.01	1769	2.7 ± 1.5	1.3 ± 0.5	3.6 ± 0.5	84/10,094
*P. campbelli* (*n* = 5)	0.26 ± 0.05	0.67 ± 0.06	0.07 ± 0.03	0.00 ± 0.01	1871	2.8 ± 1.4	1.2 ± 0.4	3.8 ± 0.5	99/11,835
F1 (*n* = 6)	0.19 ± 0.01 *	0.29 ± 0.01 *	0.35 ± 0.01 *	0.17 ± 0.01 *	3877	1.7 ± 1.4 *	2.8 ± 1.7 *	0.2 ± 0.1 *	152/17,265

^1^ SPG, spermatogonia; SER, Sertoli cells; SPTD, spermatids; P, spermatocytes at pachytene. * difference between F1 and mid-parental value is significant (Student’s *t* test, *p* < 0.001).

**Table 2:** Total length of the autosome synaptonemal complexes (SC) and the number of MLH1 foci (mean ± SD) in all autosomes in *P. sungorus, P. campbelli* and their F1 hybrids.

Group	Sex	*n* Animals	*n* Cells	SC Length, µm	MLH1 Focus Number
*P. sungorus*	f	3	119	204.7 ± 31.5	19.2 ± 2.2
	m	3	120	119.9 ± 9.5	19.1 ± 3.3
*P. campbelli*	f	3	151	190.2 ± 40.7	18.5 ± 3.6
	m	3	89	127.8 ± 12.0	19.2 ± 2.5
F1	f	4	86	176.2 ± 29.7	17.9 ± 3.6
	m	2	177	133.3 ± 15.1	19.7 ± 2.4

**Table 3:** Frequency ± SD of synaptic abnormalities of the sex chromosomes in *P. sungorus*, *P. campbelli* and their F1 hybrids. The number of animals in each group is the same as in Table 2.

Group	XY Bivalent				XX Bivalent		
	*n* Cells	Asynapsed	Synapsed without MLH1	Synapsed with MLH1	*n* Cells	Centromere Misalignment	Asynapsed Xp
*P. sungorus*	191	0.11 ± 0.02	0.36 ± 0.04	0.53 ± 0.04	151	0.52 ± 0.04	0.12 ± 0.02
*P. campbelli*	152	0.09 ± 0.02	0.26 ± 0.04	0.65 ± 0.04	321	0.37 ± 0.03	0.10 ± 0.02
F1	213	0.78 ± 0.03	0.12 ± 0.02	0.01 ± 0.02	162	0.70 ± 0.04	0.62 ± 0.04

## Supplementary Materials

The following are available online at http://www.mdpi.com/2073-4425/9/5/227/s1, File S1. This file contains the raw data, Table S1. Percentage of aberrant spermatozoa in smears of the males of *P. sungorus*, *P. campbelli* and their F1 hybrids, Table S2. Number of pachytene cells with synaptic abnormalities of the autosomes in *P. sungorus*, *P. campbelli* and their F1 hybrids, Table S3. SC length, centromeric index and number of MLH1 foci (mean ± SD) per bivalent of the identifiable chromosomes in *P. sungorus*, *P. campbelli* and F1 hybrids, Figure S1. Epididymal smears of *P. sungorus* (a) *P. campbelli* (b) and F1 hybrids (c–d). Panel e shows various sperm abnormalities of F1 hybrids, Figure S2. Sequential substages of meiotic prophase in *P. sungorus* after immunolocalization of SYCP3 (green) and γH2A.X (red). (a) leptotene, (b) zygotene, (c) early pachytene, (d) late pachytene, (e) diplotene, Figure S3. Fragments of pachytene oocytes of *P. campbelli* showing interlocking (a) and F1 hybrid showing non-homologous synapsis of autosomes (b) after immunolocalisation of SYCP3 (red) and interpretative diagrams (c,d), Figure S4. Pachytene oocyte of *P. campbelli* after c of SYCP3 (red) and centromeric proteins (blue) (a) and FISH with X painting probe of the golden hamster (green) (b).
