# Supplementary material for: Chromosome Synapsis and Recombination in Male-Sterile and Female-Fertile Interspecies Hybrids of the Dwarf Hamsters (Phodopus, Cricetidae)
Source: Genes (Basel). 2018 Apr 25;9(5):227. doi: 10.3390/genes9050227 (PMC5977167; doi:10.3390/genes9050227)
Supplement: Supplementary file 1 [file genes-09-00227-s001.zip › genes-281555 - supplementary.docx]

Supplementary Materials

**Table S1.** Percentage of aberrant spermatozoa in smears of the males of *P.* *sungorus*, *P. campbelli* and their F1 hybrids.

| **Group** | **Type of abnormality** | | | | | **N of**  **spermatozoa** |
| --- | --- | --- | --- | --- | --- | --- |
| **(n of animals)** | **Head** | **Acrosome** | **Neck** | **Tail** | **Multiple** |  |
| *P.* *sungorus* (n=5) | 1.3±0.3 | 26.1±7.5 | 1.1±0.5 | 2.0±0.8 | 2.4±1.0 | 1568 |
| *P. campbelli* (n=5) | 1.1±0.6 | 27.9±4.7 | 2.4±0.8 | 1.0±0.7 | 3.0±1.8 | 1062 |
| F1 (n=8) | 1.6±1.0 | 0.0±0.0 | 0.0±0.0 | 0.0±0.0 | 98.4±1.0 | 589 |

**Table S2.** Number of pachytene cells with synaptic abnormalities of the autosomes in *P. sungorus*, *P. campbelli* and their F1 hybrids

| **Group** | **Sex** | **n** | **Number of cells with** | | | **N cells** |
| --- | --- | --- | --- | --- | --- | --- |
|  |  | **animals** | **heterosynapsis** | **interlocking** | **associations with**  **sex chromosomes** | **examined** |
| *P. sungorus* | f | 5 | 0 | 3 | 0 | 283 |
| *P. campbelli* | f | 4 | 3 | 12 | 0 | 371 |
| F1 | f | 6 | 2 | 9 | 2 | 357 |
| *P. sungorus* | m | 3 | 0 | 1 | 0 | 347 |
| *P. campbelli* | m | 3 | 0 | 0 | 0 | 340 |
| F1 | m | 4 | 1 | 2 | 1 | 353 |

**Table S3.** SC length, centromeric index and number of MLH1 foci (mean ± S.D.) per bivalent of the identifiable chromosomes in *P. sungorus*, *P. campbelli* and F1 hybrids. The number of animals in each group is the same as in Table 2

| **Chromosome** | **Group** | **Sex** | **N cells** | **SC length, µm** | **Centromeric index** | **MLH1 foci number** |
| --- | --- | --- | --- | --- | --- | --- |
| 1 | *P. sungorus* | f | 115 | 29.8±8.2 | 0.40±0.03 | 2.0±0.6- |
|  |  | m | 119 | 17.6±1.7 | 0.43±0.02 | 2.2±0.7- |
|  | *P. campbelli* | f | 138 | 29.9±6.6 | 0.39±0.03 | 2.0±0.8 |
|  |  | m | 88 | 18.7±2.8 | 0.42±0.02 | 2.2±0.5 |
|  | F1 | f | 85 | 27.8±6.0 | 0.39±0.03 | 2.2±0.8 |
|  |  | m | 175 | 19.3±2.9 | 0.42±0.03 | 2.2±0.6 |
| 4 | *P. sungorus* | f | 115 | 23.2±6.5 | 0.36±0.03 | 2.0±0.5 |
|  |  | m | 119 | 13.8±1.3 | 0.39±0.03 | 2.0±0.6 |
|  | *P. campbelli* | f | 138 | 23.8±5.7 | 0.36±0.04 | 1.8±0.7 |
|  |  | m | 89 | 15.0±1.6 | 0.39±0.04 | 1.9±0.6 |
|  | F1 | f | 85 | 20.9±4.0 | 0.36±0.03 | 1.8±0.8 |
|  |  | m | 175 | 15.2±2.3 | 0.39±0.03 | 2.1±0.5 |
| 5 | *P. sungorus* | f | 119 | 20.2±5.4 | 0.14±0.03 | 1.8±0.6 |
|  |  | m | 120 | 13.2±1.3 | 0.15±0.02 | 1.7±0.6 |
|  | *P. campbelli* | f | 154 | 20.6±4.7 | 0.14±0.03 | 1.6±0.8 |
|  |  | m | 91 | 13.9±1.6 | 0.15±0.02 | 1.7±0.6 |
|  | F1 | f | 87 | 18.7±3.4 | 0.15±0.03 | 1.7±0.8 |
|  |  | m | 177 | 14.3±2.3 | 0.16±0.04 | 1.8±0.6 |
| 7 | *P. sungorus* | f | 119 | 13.9±3.8 | 0.29±0.04 | 1.3±0.6 |
|  |  | m | 120 | 10.0±0.9 | 0.31±0.03 | 1.4±0.6 |
|  | *P. campbelli* | f | 153 | 14.7±3.1 | 0.29±0.03 | 1.4±0.6 |
|  |  | m | 89 | 10.3±1.3 | 0.30±0.04 | 1.4±0.5 |
|  | F1 | f | 87 | 13.4±2.8 | 0.30±0.03 | 1.3±0.7 |
|  |  | m | 177 | 10.4±1.5 | 0.30±0.04 | 1.4±0.6 |
| X | *P. sungorus* | f | 72 | 12.4±3.5 | 0.39±0.05 | 0.9±0.3 |
|  |  | m | 106 | 12.6±1.2 | 0.20±0.04 | 0.6±0.5 |
|  | *P. campbelli* | f | 131 | 13.0±3.5 | 0.43±0.05 | 0.7±0.5 |
|  |  | m | 86 | 14.8±1.6 | 0.25±0.05 | 0.7±0.5 |
|  | F1 | f | 61 | 12.2±2.7 | 0.40±0.06 | 0.9±0.3 |
|  |  | m | 38 | 13.5±2.3 | 0.24±0.07 | 0.4±0.5 |

**
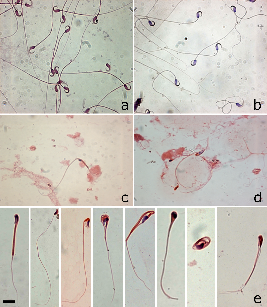
**

**Figure S1**. Epididymal smears of *P. sungorus* (a) *P. campbelli* (b) and F1 hybrids (c-d). Panel e shows various sperm abnormalities of F1 hybrids. Bar: 10 µm.

**
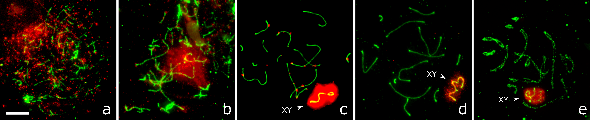
**

**Figure S2**. Sequential substages of meiotic prophase in *P. sungorus* after immunolocalization of SYCP3 (green) and γH2A.X (red). Bar: 10 µm. (a) leptotene, (b) zygotene, (c) early pachytene, (d) late pachytene, (e) diplotene.

**
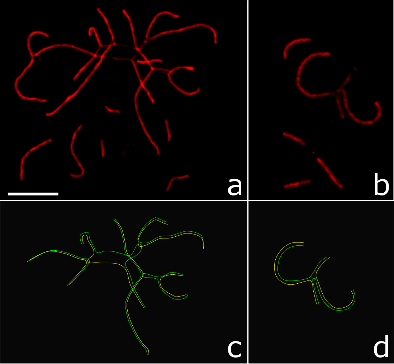
**

**Figure S3.** Fragments of pachytene oocytes of *P. campbelli* showing interlocking (a) and F1 hybrid showing non-homologous synapsis of autosomes (b) after immunolocalisation of SYCP3 (red) and interpretative diagrams (c, d). Bar: 10 µm.


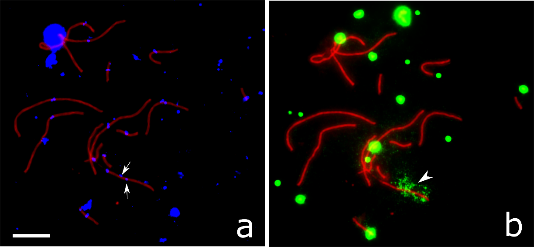


**Figure S4.** Pachytene oocyte of *P. campbelli* after immunolocalisation of SYCP3 (red) and centromeric proteins (blue) (a) and FISH with X painting probe of the golden hamster (green) (b)*.* Arrow shows Xq. Bar: 10 µm.
